# Cellular content plays a crucial role in Non‐typeable *Haemophilus influenzae* infection of preinflamed *Junbo* mouse middle ear

**DOI:** 10.1111/cmi.12960

**Published:** 2018-10-15

**Authors:** Pratik P. Vikhe, Tom Purnell, Steve D.M. Brown, Derek W. Hood

**Affiliations:** ^1^ Mammalian Genetics Unit MRC Harwell Institute Harwell Campus Oxfordshire OX11 0RD UK

## Abstract

Non‐typeable *Haemophilus influenzae* (NTHi) is a major pathogen causing acute otitis media (AOM). The relationship between the cellular content of the middle ear fluid (MEF) during AOM and infection of NTHi is poorly understood. Using the *Junbo* mouse, a characterised NTHi infection model, we analysed the cellular content of MEF and correlated the data with NTHi titres. The MEF of the *Junbo* mouse was heterogeneous between ears and was graded from 1 to 5; 1 being highly serous/clear and 5 being heavily viscous/opaque. At seven‐day post‐intranasal inoculation, NTHi was not found in grade‐1 or 2 fluids, and the proportion of MEF that supported NTHi increased with the grade. Analyses by flow cytometry indicated that the cellular content was highest in grade‐4 and 5 fluids, with a greater proportion of necrotic cells and a low‐live cell count. NTHi infection of the middle ear increased the cell count and led to infiltration of immune cells and changes in the cytokine and chemokine levels. Following NTHi inoculation, high‐grade infected MEFs had greater neutrophil infiltration whereas monocyte infiltration was significantly higher in serous noninfected low‐grade fluids. These data underline a role for immune cells, specifically monocytes and neutrophils, and cell necrosis in NTHi infection of the *Junbo* mouse middle ear.

## 1 INTRODUCTION

Otitis media (OM) is characterised by inflammation of the middle ear and is the most common cause of hearing impairment in children (MacArthur, Hausman, Kempton, Lighthall, & Trune, 2012). Based on variables such as the chronicity of inflammation and the state of middle ear (bulla) fluid, OM is classified into different categories (Myburgh, van Zijl, Swanepoel, Hellström, & Laurent, 2016). A common form is acute suppurative OM, typically caused by bacterial infection; the most common associated bacteria are nontypeable *Haemophilus influenzae* (NTHi), *Streptococcus pneumoniae,* and *Moraxella catarrhalis* (Hood et al., 2016). Interplay between various immune components in the middle‐ear cavity and the bulla fluid play a significant part in the onset of disease and subsequent eradication of bacteria (Hernandez et al., 2015). The middle ear cavity is lined with a mucosal membrane containing secretory glands that produce major components of both mucoid and serous fluids (Orita et al., 2002). Serous fluid is considered to aid mucociliary clearance, and mucoid material provides lubrication to the epithelial cell boundary, thus, acting as the first line of innate immune defence. The anatomy of the middle ear also plays a significant part in the initial clearance of infecting bacteria. In infants and young children, the arrangement of the Eustachian tube is shorter, wider, and more horizontal compared with adults, making it more difficult to attain successful mucociliary clearance (Massa, Lim, Kurono, & Cripps, 2015). A sustained presence of bacteria and/or bacterial antigens can stimulate secretory cells and lead to changes in the middle‐ear fluid composition during the exacerbation of OM.

OM reoccurs in 20–30% of the paediatric population (Roos, Hakansson, & Holm, 2001; Veenhoven et al., 2003), and the pathophysiology behind this is still not clearly understood. Persistence of bacteria/virus/bacterial material in the middle ear might initiate the inflammatory response that leads to recurrence of OM, and the cellular content of the bulla fluid is likely a significant factor that influences clearance (Forséni, et al., 1999). The chinchilla model for OM has indicated a significant role for NTHi biofilm in the middle ear in the modulation of the host cellular response (Sato, Liebeler, Quartey, Le, & Giebink, 1999). Experimental induction of OM in rat and mouse by direct inoculation of bacteria into the noninflamed middle ear causes a rapid cellular response typified by a large increase in granulocytes, macrophages, and a mild increase in the lymphocyte populations (Forséni, et al., 1999; Hernandez et al., 2015). The pathological chronicity of OM increases during the long‐lasting or reoccurring infection in the middle ear. The dynamics of the cellular response against the infecting bacteria is still unclear. In the current study, using a *Junbo* mouse model of OM, we analysed the correlation between the cellular content of the middle‐ear fluid against the ability of NTHi to infect the preinflamed middle ear. The *Junbo* mouse spontaneously develops chronic middle‐ear inflammation under specific pathogen‐free conditions (Parkinson et al., 2006). Earlier studies have shown that intranasal inoculation of the *Junbo* mouse with NTHi typically results in 80% of the middle ears being infected (Hood et al., 2016). Thus, 20% of *Junbo* middle ears are not subsequently infected by NTHi. We show that the type of immune cell and its functional state both contribute to the NTHi infection observed in the inflamed middle ear of *Junbo* mice.

## 2 RESULTS

### 2.1 Variation in the bulla fluid influences NTHi infection

**Figure 1:**
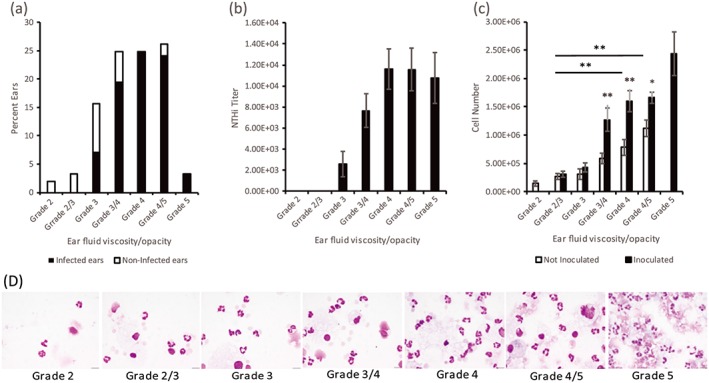
Variation in *Junbo* mouse bulla fluid. (a) The bulla fluid was graded from 1 to 5 on the basis of its appearance, 1 being most serous/clear and 5 being most viscous/opaque (all bars). (b) Average bacterial titers per μl for the respective ear fluid grades post‐7 days intranasal inoculation of NTHi (filled bars). (c) Total cell count for the different grades of ear fluid in noninoculated (empty bars) and inoculated (filled bars) *Junbo* mice (*P < 0.05, **P < 0.01). (d) Cyto‐spun and giemsa stained images of different grade middle ear fluids (Scale bar: 20 μm)

The *Junbo* mouse spontaneously develops fluid in the middle ear (Parkinson et al., 2006) and a single intranasal inoculation with NTHi results in infection in a majority of middle ears; ears with no bulla fluid do not support NTHi growth (Hood et al., 2016). Around 80% of the middle ears with fluid in the *Junbo* mouse are NTHi culture positive at 7 days postinoculation with NTHi 162 sr. Even although these mice were genetically similar and were bred under specific pathogen free conditions, 20% of the middle ears were not infected with the NTHi. To investigate this variability, we sought to understand the correlation between bulla fluid and NTHi infection load. An initial general observation was the variation in the quality of the bulla fluid, from being serous to highly mucoid and pus like; similar observations are noted in human patients (Carrie, Hutton, Birchall, Green, & Pearson, 1992). On this basis, the *Junbo* bulla fluid was graded from 1 to 5, wherein 1 being the most serous/clear and 5 being the most viscous/opaque. Middle‐ear fluids, which were deemed intermediate between two grades, or contained mixed aliquots of two grades of fluid, were described as intermediate grades 2/3, 3/4, and 4/5. Across 153 middle ears that contained fluid and were sampled, nearly 5% of the bulla fluids were of serous type grade 2 or 2/3, 41% were more dense/slightly viscous grade 3 or 3/4, 51% were pus filled/viscous grade 4 or 4/5, and 3% were very viscous/opaque grade 5 (Figure 1a). Grade 1 highly serous/clear fluids were rarely observed, thus, not included in current analyses.

When overlaying this middle‐ear fluid characterisation with the NTHi infection data, 79% of all middle ears were infected with NTHi, and 21% of middle‐ear fluids were not infected. A clear correlation between NTHi infection and viscosity of the fluid was observed (Figure 1a). The *Junbo* mouse middle ears with grade 2 and 2/3 serous fluid had no NTHi infection (0%) present at seven‐day postinoculation, whereas 46% of grade 3, 79% of grade 3/4, 100% of grade 4, and 92% of grade 4/5 fluids were infected. All middle ears with highly viscous grade‐5 fluids cultured NTHi at seven‐day postinoculation; grade 5 fluid was generally not found in noninoculated mice. The NTHi titres also correlated with the grade of middle‐ear fluid (Figure 1b); the more serous fluids had low‐bacterial titres and as the viscosity increased so did the average NTHi titre. *Junbo* mouse middle ears with the most serous grade‐1 fluid were found on rare occasions, and no NTHi were present at seven‐day postinoculation. Thus, a direct correlation has been shown between the nature of the bulla fluid and the NTHi bacterial load in the middle ear of the *Junbo* mouse.

Having established the direct relationship between the viscosity/opacity of the *Junbo* bulla fluids and the NTHi bacterial load in the middle ear, next, we examined the total cell count in the bulla fluids as this would seem a logical factor to contribute to the different fluid types and associated survival of NTHi. Significant difference in total cell count was observed in the different grades of fluid (Figure 1c). The high‐viscosity grade 4 and 4/5 fluids had a total cell count of 9.12 × 10^5^ and 1.27 × 10^6^ cells/μl respectively in noninoculated mice, which was significantly higher when compared with the serous grade 2, 2/3, and 3 fluids, with a total cell count of 1 × 10^5^, 3.14 × 10^5^, and 4.01 × 10^5^ cells/μl, respectively. The difference in cell number for different grades of fluid was confirmed by giemsa staining (Figure 1d). To ascertain whether the presence of the NTHi alters the cell number, the cell count was performed on the bulla fluids obtained seven‐day postinoculation with NTHi. The total cell count in the serous grade 2/3 fluid (negative for NTHi culture) post‐NTHi inoculation was not significantly changed whereas the total cell count significantly increased in grade‐4 fluid (positive for NTHi culture), attaining an average cell count of 1.89 × 10^6^ cells/μl, consistent with an infiltration of cells in the presence of NTHi.

### 2.2 NTHi infects *Junbo* mouse bulla fluids with necrotic cell population

**Figure 2:**
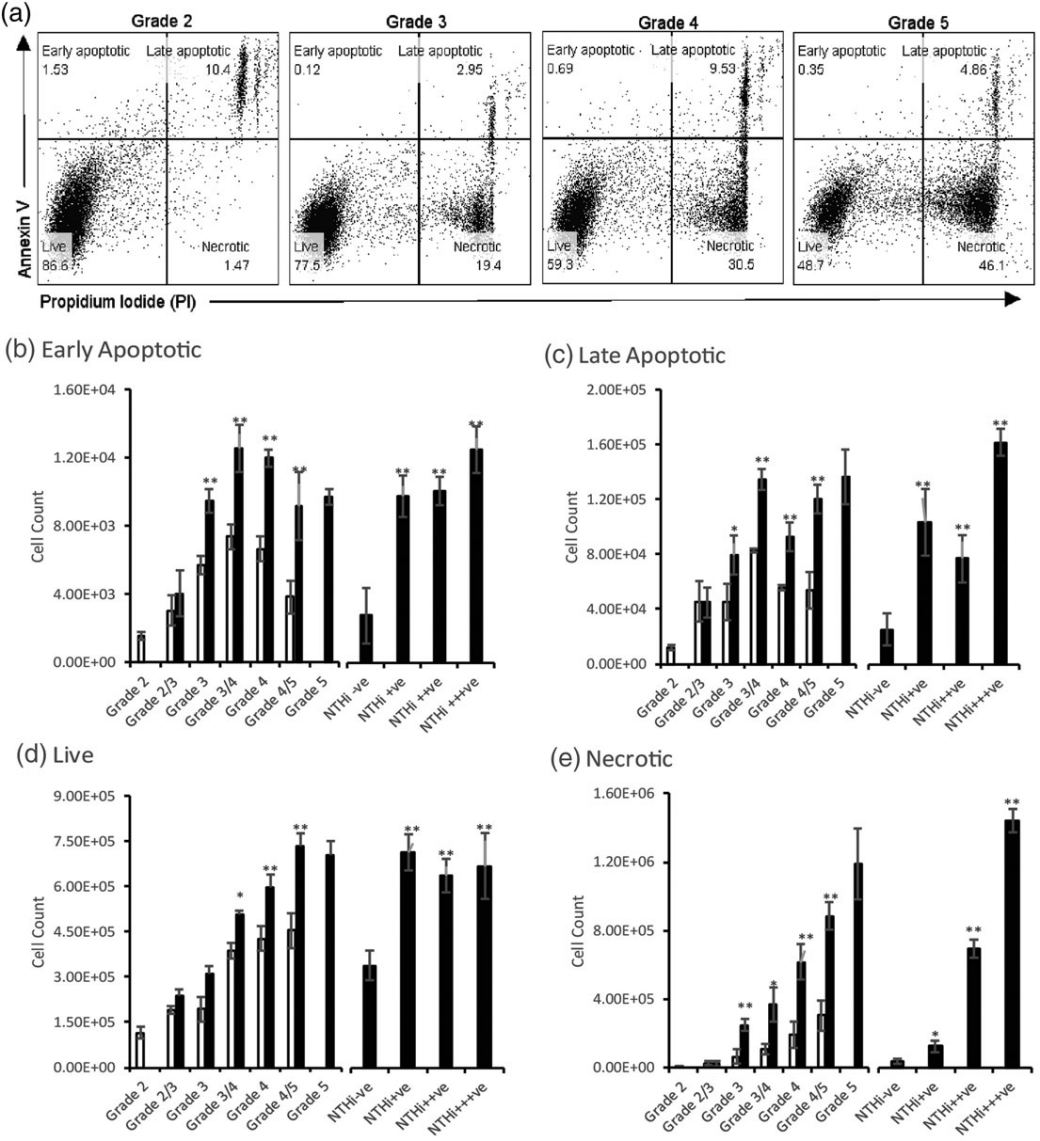
Variation in viable cell content in *Junbo* mouse bulla fluid. (a) Gating strategy applied to differentiate live (Annexin V–ve, PI‐ve), early apoptotic (Annexin V + ve, PI‐ve), late apoptotic (Annexin V + ve, PI+ve), and necrotic (Annexin V‐ve, PI+ve) cell content in *Junbo* mouse bulla fluids. The volume of ear fluids from different grades was adjusted by FACS buffer, such that constant cell numbers were stained for flow cytometry and 50,000 single cell events were counted. Early apoptotic (b), Late apoptotic (c), Live (d) and Necrotic (e) cell count calculated from total cell count and flow‐cytometry percentages across different grades of ear fluid from non‐inoculated (empty bars) and inoculated (filled bars) *Junbo* mice. NTHi−ve–no NTHi, NTHi+ − 1 to 10^2^ CFU/μl, NTHi++ − 10^2^ to 10^3^ CFU/μl, NTHi+++ − 10^3^ to 10^4^ CFU/μl. *n* = 13, *P < 0.05,**P < 0.01

It would appear somewhat counter‐intuitive that despite the higher cell count in high‐viscosity fluids, NTHi is able to exist effectively in these middle ears; we, thus, investigated the viability state of these cell populations. Using flow cytometry analysis of the middle‐ear fluids collected from NTHi 162 sr inoculated and noninoculated *Junbo* mice, we determined the proportion of apoptotic and necrotic cells based on the Annexin V and propidium iodide binding (Figure 2a). The serous fluids had high proportions of live cells with low‐necrotic cell proportions, whereas high‐viscosity grade fluids had low proportions of live cells with high proportions of necrotic cells. There was no significant variation in the apoptotic cell proportions across the different grade of fluids. NTHi infection of the middle ear significantly increased live, necrotic, and apoptotic cells in the high‐viscosity (NTHi+ve) middle‐ear fluids.

The proportions obtained from the above analysis were applied to the actual cell count for each middle‐ear fluid type, and significant variation was seen in the number of apoptotic, necrotic, and live cells. In the noninoculated *Junbo* mice, the serous grade‐2 fluids had 1.5 × 10^3^ early apoptotic cells/μl and high‐viscosity grade‐4 fluids 3 × 10^3^ early apoptotic cells/μl (Figure 2b). The late apoptotic cell population did not differ from the early apoptotic cell population in the case of grade‐2 fluids but a 10‐fold increase was observed in grade‐4 fluids taking the average count to 5 × 10^4^ cells/μl (Figure 2c). The inoculation of NTHi in the mouse did not change the apoptotic cell count in grade 2/3 fluids, but a significant increase was seen in the higher viscosity grade fluids. On comparing the apoptotic cell count in the NTHi culture negative (NTHi−ve) with NTHI culture positive NTHi+ve ear fluids postinoculation, there was an increase from 2 × 10^3^ to 1.5 × 10^4^ cells/μl for early apoptotic cells. In the case of late apoptotic cells, the cell count increased from 2 × 10^4^ to 1.6 × 10^5^ cells/μl in the presence of NTHi, suggesting that NTHi stimulates host cell apoptosis in the *Junbo* middle ear.

The serous grade‐2 fluids had 1–2 × 10^5^ live cells/μl that comprised nearly 70% of total cells in that grade of fluid. Grade‐4 fluid had 5 × 10^5^ live cells/μl that comprised only 40% of total cells in that grade of fluid (Figure 2d). Thus, the serous middle‐ear fluid had a higher proportion of live cells compared with high‐viscosity fluids. NTHi inoculation did not change the live cell number in the grade 2/3 fluids, but it was significantly increased in the high‐viscosity grade fluids. The comparative analysis of live cells between NTHi+ve and NTHi−ve inoculated *Junbo* mouse middle‐ear fluids showed that the presence of NTHi stimulated a significant increase in the live cell numbers, from 3 × 10^5^ to 7 × 10^5^ cells/μl (Figure 2d).

The necrotic cell number was significantly lower in the case of grade‐2 fluid (1 × 10^5^ cells/μl, which was only 5% of total cells) compared with the viscous grade fluids (3–6 × 10^5^ cells/μl, which was 55% of total cells; Figure 2e). The increase in the necrotic cell number across the ear fluid correlated with increase in viscosity and grade of ear fluid, suggesting that it might be one of the major contributing factors to the variation observed in ear fluid property. Following NTHi inoculation, no significant change in necrotic cell number was seen in the grade 2/3 fluids, but it significantly increased across the higher viscosity grade fluids. Comparative analysis of the NTHi−ve with NTHi+ve inoculated ear fluids showed a significant increase in the necrotic cells (from 2.5 × 10^5^ to 1.1 × 10^6^ cells/μl) as the NTHi titre increased, thus, indicating the clear association between NTHi infection and high‐necrotic cell content.

### 2.3 Immune cell dynamics play a crucial role in NTHi infection of *Junbo* mouse bulla fluids

**Figure 3:**
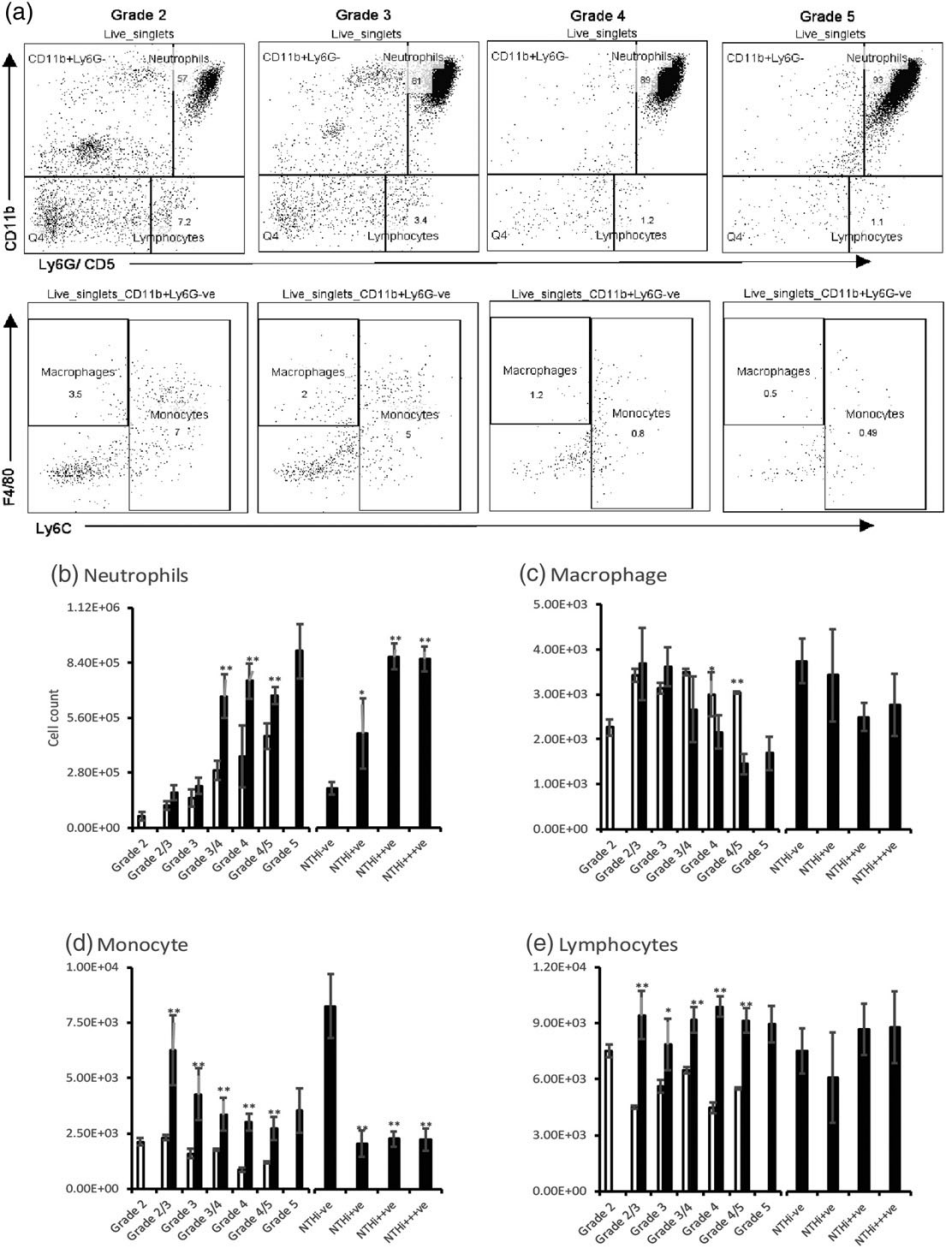
Variation in immune cell content in *Junbo* mouse bulla fluid. (a) Gating strategy applied to differentiate live cells (sytox green‐ve), neutrophils (CD11b + veLy6G + ve), macrophages (CD11b + veF4/80 + veLy6G‐veLy6C‐ve), monocytes (CD11b + veLy6C + Lvey6G‐veF4/80 ± ve), and lymphocytes (CD11b‐veCD5 + ve) present in *Junbo* mouse bulla fluids. The volume of ear fluids was adjusted with FACS buffer such that constant cell numbers were stained for flow cytometry and 50,000 live single cell events were counted. Neutrophil (b), macrophage (c), monocyte (d), and lymphocyte (e) cell counts were calculated from total cell count and flow‐cytometry percentages across different grades of ear fluid from noninoculated (empty bars) and inoculated (filled bars) *Junbo* mice. NTHi−ve–no NTHi, NTHi+ − 1 to 10^2^ CFU/μl, NTHi++ − 10^2^ to 10^3^ CFU/μl, NTHi+++ − 10^3^ to 10^4^ CFU/μl. *n* = 12, *P < 0.05,**P < 0.01

Intranasal inoculation of NTHi significantly increased the live and necrotic cell numbers in the high‐viscosity middle‐ear fluids after 7 days, and these fluids were positive for NTHi culture. In contrast, in the serous grade fluids, there was no significant difference in the cell number, and these fluids were negative for NTHi culture. Thus, the presence of NTHi affected the number of cells suggesting that there was an associated infiltration of cells into the middle ear cavity. To determine the type of immune cells infiltrating the middle‐ear cavity post‐NTHi infection, the level of neutrophils (CD11b + Ly6G+), macrophages (CD11b + F4/80 + Ly6G‐Ly6C‐), monocytes (CD11b + Ly6C + Ly6G), and lymphocytes (CD11b‐CD5+) was determined in the inoculated and noninoculated *Junbo* middle‐ear fluids using flow cytometry (Figure 3a).

**Figure 4:**
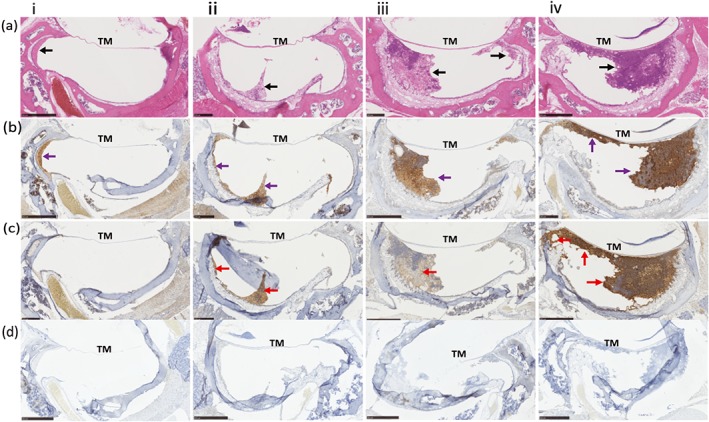
Immunostaining of NTHi inoculated *Junbo* mouse middle ears. Formalin fixed paraffin embedded sections of four NTHi inoculated *Junbo* mouse middle ears (i,ii,iii,iv) with variable volumes and grades of ear fluid (a) Haematoxylin and Eosin‐stained section of middle‐ear bulla, showing the tympanic membrane and the middle‐ear fluid—black arrow. (b) DAB immunostaining of neutrophils in the middle‐ear fluid with rabbit anti‐MPO antibody showing presence in the fluid—purple arrow. (c) DAB immunostaining of NTHi 162 sr in the middle ear fluid with anti‐NTHi162 antibody showing the presence and uneven distribution of bacteria across the fluid—ense patch indicated by red arrow. (d) Staining using control rabbit serum (non‐immune) as primary antibody and rabbit VERTEX ABC kit for detection

Neutrophils were predominant across all grades of middle‐ear fluid (Figure 3b) but were significantly higher in number in high‐viscosity grade 4/5 fluids (4.65 × 10^5^ cells/μl; this comprised 90% live cells) compared with serous grade 2‐fluid (5.8 × 10^4^ cells/μl; this comprised 65% live cells). Following NTHi inoculation, the neutrophil numbers did not change in low‐grade 2 and 2/3 fluids but significantly increased from an average of 3 × 10^5^ to 6 × 10^5^cells/μl in higher viscosity grade 3/4, 4, and 5 fluids. The NTHi−ve ear fluids from inoculated *Junbo* mice had significantly lower neutrophil content compared with NTHi+ve ear fluids, likely indicative of constant neutrophil infiltration in response to the presence of NTHi. Immunohistochemistry analysis (Figure 4) showed that the NTHi was predominantly present in neutrophil‐rich middle‐ear fluids and was unevenly distributed in these fluids; the high density observed in some portions of fluid might be consistent with the biofilm associated with neutrophil extracellular traps that is observed with OM in the middle ear of the chinchilla (Jurcisek & Bakaletz, 2007).

Macrophage numbers (Figure 3c) did not significantly vary across the grades of ear fluid but were somewhat higher in serous grade‐2 fluids compared with viscous grade‐4 and 4/5 fluids. NTHi inoculation slightly increased the macrophage number in serous grade‐2/3 fluids but significantly decreased it in the high‐viscosity grade‐4/5 fluids; from 3 × 10^3^ cells/μl in noninoculated to 1.4 × 10^3^ cells/μl in inoculated mice. A slight reduction in macrophage number was observed in NTHi+ve compared with NTHi−ve ear fluids, but this was not statistically significant.

Monocyte number (Figure 3d) was relatively constant across all grades of ear fluid from noninoculated *Junbo* mice, but it significantly increased following NTHi inoculation. In serous grade‐2/3 fluids, monocyte number increased nearly threefold, from 2.3 × 10^3^ cells/μl to 6.2 × 10^3^ cells/μl that was 7% of live cells, post‐NTHi inoculation, and one major observation noted was that all the ear fluids (post‐NTHi inoculation) were NTHi−ve. The comparative analysis between NTHi+ve and NTHi−ve middle‐ear fluids showed a significant decrease in the monocyte count in the fluids that were culture positive for NTHi; it was constant at 2.5 × 10^3^ cells/μl (comprising 0.5 to 2% of live cells) across all the NTHi+ve fluids, irrespective of the NTHi titre. These observations indicate association between monocytes and NTHi load in the *Junbo* mouse middle ear.

The number of lymphocytes observed (Figure 3e) showed no significant difference across the grades of fluid, but NTHi inoculation significantly increased the lymphocyte count. The presence of NTHi in the bulla fluid at day 7 of post‐inoculation did not affect the lymphocyte count that remained between 8 and 9 × 10^3^ cells/μl. These observations suggest that the presence of NTHi in high‐viscosity fluids leads to a continuous infiltration of neutrophils, whereas, there is an increase in monocytes in serous fluids that were NTHi negative postinoculation. Thus, these data indicate the significance of the dynamic between neutrophil and monocyte infiltration in the ability of NTHi to cause long‐term infection in the *Junbo* mouse middle ear.

### 2.4 NTHi infection in preinflamed middle ear causes changes in cytokine and chemokine levels

**Figure 5:**
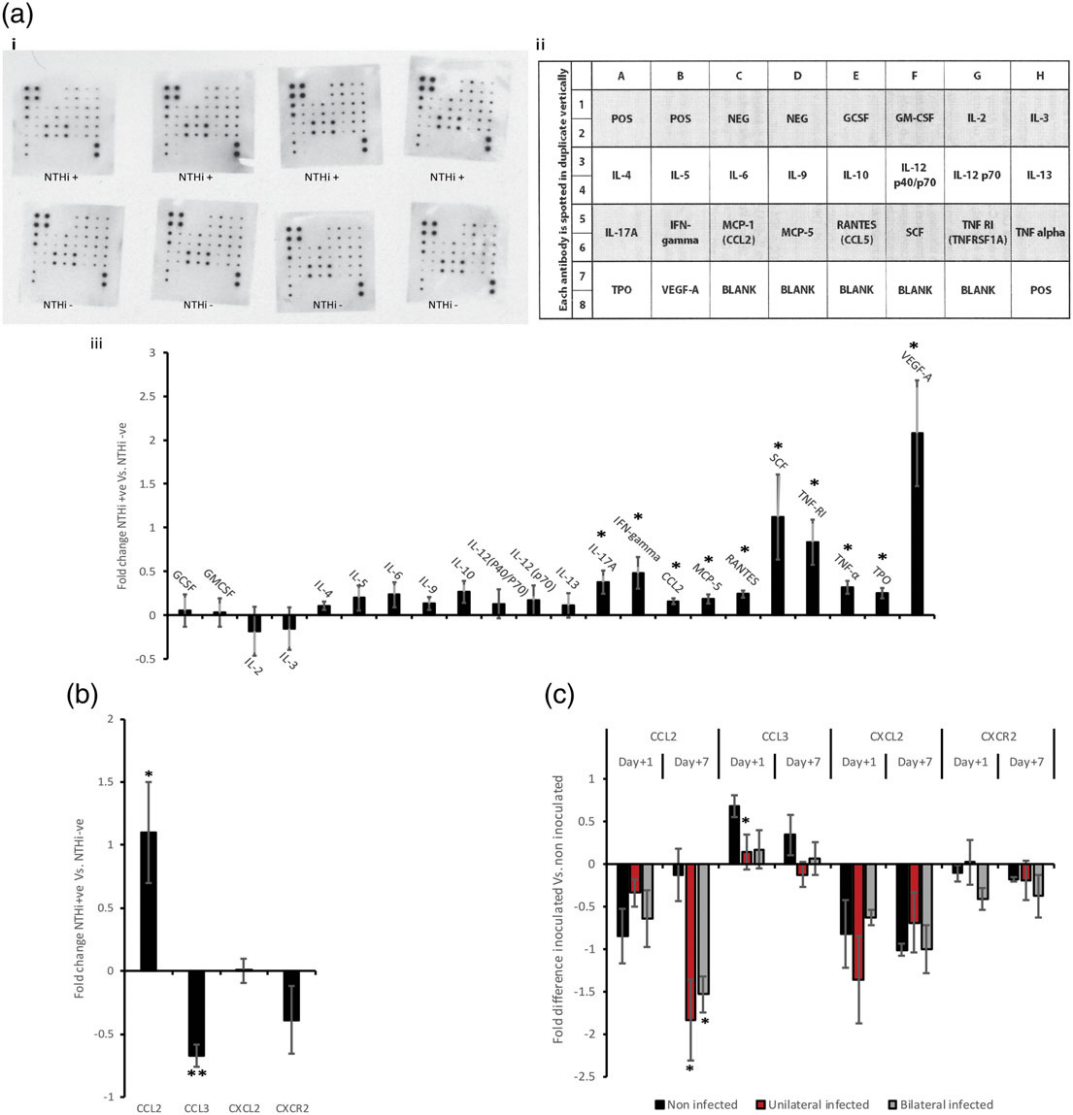
Cytokine and chemokine levels in *Junbo* mouse bulla fluid post‐NTHi inoculation. (a) i. Immunoblot of cytokine and chemokine levels in *Junbo* mouse bulla fluid with and without NTHi infection 7 days after intranasal inoculation. ii. Layout of dot blot pattern (RayBiotech) on the immunoblot used for cytokine and chemokine analysis. iii. Fold changes in NTHi+ve bulla fluid cytokine and chemokine levels compared with NTHi−ve bulla fluids calculated from the immunoblots using Image Lab (BioRad^tm^; *n* = 4). (b) Fold changes in NTHi+ve bulla fluid cytokine and chemokine levels in comparison with NTHi−ve bulla fluid obtained by ELISA (*n* = 5). (c) Fold changes in serum cytokine and chemokine levels from NTHi inoculated *Junbo* mice compared with serum from noninoculated *Junbo* mice obtained by ELISA (*n* = 6). *P < 0.05

The *Junbo* mouse, a chronic OM model, has a highly inflamed and hypoxic environment in the middle ear (Cheeseman et al., 2011). After intranasal inoculation of the *Junbo* mouse by NTHi, the cytokines present in the middle‐ear fluid of NTHi+ve and NTHi−ve ear fluids were profiled at day 7 of postinoculation (Figure 5). The levels of granulocyte colony‐stimulating factor (GCSF), granulocyte‐macrophage colony‐stimulating factor, and interleukins—2,3,4,5,6,9,10,12, and 13—did not significantly vary following NTHi infection in the *Junbo* mouse middle‐ear fluid (Figure 5a). Major Th cell‐dependent cytokines, IL‐17 and INF‐gamma (Lucey, Clerici, & Shearer, 1996), significantly increased in middle‐ear fluids with NTHi infection. The leukocyte chemotactic chemokines CCL2, MCP‐5, and RANTES were also significantly increased in the NTHi‐infected middle‐ear fluids. Other cytokines, such as stem cell factor (SCF) and Thrombopoietin (TPO) that alter haematopoietic stem cell differentiation (Da Silva & Frossard, 2005; Lukacs et al., 1996; Lupia, Goffi, Bosco, & Montrucchio, 2012a), were significantly elevated in the presence of NTHi. Tumor necrosis factor alpha (TNF‐α) and vascular endothelial growth factor (VEGF‐A), major signalling proteins that alter and recruit neutrophils, were significantly increased in NTHi‐infected middle‐ear fluids. VEGF‐A showed the highest, nearly twofold, increase in the NTHi‐infected middle‐ear fluids compared with NTHi–ve ones. Among other chemokines, CCL3 showed a significant decrease in the middle‐ear fluids with NTHi present (Figure 5b). The serum levels (Figure 5c) of CCL3 were elevated at day 1 of postinoculation in *Junbo* mouse middle ears with no NTHi infection. CCL3 plays a crucial role in chemotaxis of monocytes, macrophages, and neutrophils at the site of inflammation (Baba & Mukaida, 2014). It also inhibits NTHi‐induced OM in TNF^−/−^ mice by reversing impaired macrophage‐mediated phagocytosis and killing (Leichtle et al., 2010). Our observed association between initial elevation of serum CCL3 levels and infiltration of monocytes in *Junbo* mice middle ears with no NTHi infection suggests it could play a role in defence against NTHi infection in pre‐inflamed middle ears. Other chemokines, like CXCL2 and CXCR2, did not show any significant variation in levels post‐NTHi inoculation and infection.t1

## 3 DISCUSSION

The current study investigates the nature of the bulla fluids in the *Junbo* mouse and its association with middle ear infection by NTHi, a prevalent pathogen causing AOM (Barkai, Leibovitz, Givon‐Lavi, & Dagan, 2009; Sierra et al., 2011) in children. AOM is characterised by inflammation and the formation of fluid in the middle ear (Vergison, 2008); characteristics of this fluid typically also play a significant part in defining the severity and chronicity of OM (Lin et al., 2003; Massa et al., 2015). Thus, gaining knowledge of the ear fluid pathology is important not only for better understanding the disease but also could improve our ability to combat OM. The *Junbo* mutant mouse is a useful tool in this respect; it develops middle ear inflammation and bulla fluid at around 4–5 weeks of age, and this is sustained in its later life (Hood et al., 2016). At 12 weeks, the age at which we have established robust NTHi infection seven‐day postintranasal inoculation (Hood et al., 2016), we see substantial variability in the characteristics of the *Junbo* mouse middle‐ear fluid over a range similar to that observed in human patients with OM (commonly classified as serous, mucoid, or pus filled; Furukawa, Kubo, & Yamashita, 1995). Our grading strategy was based on discernible differences observed in the viscosity and opacity of the ear fluid, and we found a direct relationship between NTHi infection and ear‐fluid grade at seven‐day postintranasal inoculation. NTHi was absent in the serous grade‐2 ear fluids whereas almost all the ears containing high ‐iscosity fluid of grades 4 and 5 had bacteria present.

A major complication that leads to the chronicity of AOM is the persistence or infection of the bacteria in an already inflamed environment of the middle ear. The infiltration of the immune cells on challenge with the pneumococcus has been studied in rat and chinchilla‐middle ears but unlike our infection model, both have no inflammation prior to inoculation (Forséni, et al., 1999; Sato et al., 1999). The *Junbo* mouse has a perturbed immune response due to the Evi1 mutation that affects NF‐kB regulation (Li Konduru et al., 2017), which disturbs middle‐ear homeostasis causing a highly inflamed middle‐ear environment and OM. The efficient establishment of NTHi infection in this highly inflamed environment with high immune‐cell content is somewhat counter‐intuitive but mimics the condition found in patients suffering from long‐term and recurrent AOM (Sharma & Pichichero, 2013). In the *Junbo* mouse post‐NTHi inoculation, it was observed that in ear fluids positive for NTHi culture that neutrophil infiltration continues, and this increase was correlated with the NTHi titre. NTHi is known to induce neutrophil extracellular trap formation (Juneau, Pang, Weimer, Armbruster, & Swords, 2011) and is able to form a biofilm in the extracellular DNA and other material released as the result of this process (Tikhomirova & Kidd, 2013); DNA and other released material may also act as nutrients to facilitate NTHi growth. Our analysis of apoptotic, necrotic, and live cells showed that the presence of NTHi significantly increased the number of apoptotic and necrotic cells present, suggesting that a similar process occurs in the middle ear of the *Junbo* mice. From our data displayed in Figure 4, NTHi likely associates with the DNA matrix released by host immune cells (biofilm‐associated form) but also exist in planktonic and host cell‐associated forms in other areas of the middle‐ear cavity.

NTHi is reported to be internalised and induce apoptosis of type II alveolar epithelial cells (Goyal, Singh, Ray, Srinivasan, & Chakraborti, 2015). The observations in the current study are consistent with the same ability of NTHi to induce apoptosis being relevant in the middle ear of the *Junbo* mouse. In the normal inflammatory response, the clearance of these apoptotic and necrotic cells is essential to avoid the perturbation of the infection (McArthur et al., 2015). Monocytes and macrophages are the cells that play a major role at this stage of the inflammation (McArthur et al., 2015). The analysis of these cell populations in the middle ear of the *Junbo* mouse showed a distinct correlation of monocyte numbers with infection of NTHi; high‐monocyte count in the serous grade fluid correlated with the low‐apoptotic and necrotic cell count with no NTHi infection. Thus, the monocyte either directly or indirectly can play a significant role in inhibiting the infection of NTHi in the middle‐ear fluid.

Numerous studies have focused on the changes in the cytokine and chemokine levels in the middle ear with respect to the onset and progression of OM (Juhn et al., 2008; Sato et al., 1999), and these signalling molecules influence the migration and functioning of the immune cells. Using the *Junbo* mouse, we have for the first time analysed the changes in the major cytokines and chemokines in response to the presence of NTHi in an already highly inflamed middle ear. The analysis showed that VEGF‐A was twofold elevated in middle‐ear fluids infected with NTHi compared with those with no NTHi infection. VEGF‐A is the major signalling molecule involved in vasodilation; it promotes cellular migration and is induced under hypoxic condition (Cheeseman et al., 2011). The hypoxic environment in the *Junbo* mouse middle ear plays a critical role in the chronicity of OM in this mouse (Cheeseman et al., 2011), and our current observation indicates its significance in NTHi infection. Another major molecule showing significant elevation was pro‐inflammatory cytokine TNF‐α; this plays a major role in stimulating neutrophil degranulation, respiratory burst, and release of platelet activating factor (PAF) (Ogura, Furukawa, Tada, Ikeda, & Yamashita, 2008). PAF induces middle‐ear inflammation (Ogura et al., 2008) and is found in higher levels in pus‐filled human middle‐ear fluids (Tachibana et al., 1996). TNF‐α was also significantly elevated in the chinchilla middle ear after pneumococcus inoculation but at a later time point (72 hrs) following infection (Sato et al., 1999). The Th1 dependent INF‐γ and Th17 dependent IL‐17 were also elevated in the middle‐ear fluids with NTHi indicating the role of a lymphocyte response to the infection. The Th (CD4+) cells are found to be the dominant lymphocyte population in the middle‐ear effusions from the young children (Skotnicka, Stasiak‐Barmuta, Hassmann‐Poznanska, & Kasprzycka, 2005); we are currently investigating the response of these cells to NTHi in the *Junbo* mouse. Chemokines like SCF and TPO were elevated in NTHi+ve middle‐ear fluids; these not only induce inflammation but also regulate stem cell function. SCF plays a major role in mast cell degranulation (Lukacs et al., 1996) and the differentiation of CD34^+^ cells into mast cells in the upper respiratory tract (Da Silva & Frossard, 2005). Similarly, TPO plays a role in haematopoietic stem cell differentiation (Lupia, Goffi, Bosco, & Montrucchio, 2012a). Thus, these two chemokines play a significant role in the increase of inflamed state seen in NTHi‐infected *Junbo* mouse middle ears. Another chemokine, CCL3, which inhibits the proliferation of immature progenitor cells but activates the proliferation of mature progenitor cells (Baba & Mukaida, 2014), was not potentiated in the NTHi infected ear fluids. The significance of CCL3 in protecting against NTHi‐induced OM in mice is already known (Deniffel et al., 2017), and our data on CCL3 levels in both middle‐ear fluids and serum suggests its role in inhibiting the infection of NTHi. Another important chemokine, CCL2, was elevated in the NTHi‐infected mice. CCL2 is known to contribute to inner ear inflammation stimulated by NTHi in the mouse (Woo, Pan, Oh, Lim, & Moon, 2010) and is an important mediator in the recruitment of monocytes; this consequently affects the cellular response (Qian et al., 2011; Raghu et al., 2017). Thus, in the *Junbo* mouse middle ear, CCL2 is also playing an important part in mediating the cellular response against NTHi.

The current study indicates that the cellular content is a significant factor in the variability observed in the middle‐ear fluid in the *Junbo* mouse, and this affects the ability of NTHi to infect; the reason(s) underlying the variability in the ear fluids remain unclear. In humans also, the chronicity of OM is often associated with the differences observed in the ear fluids (Carrie et al., 1992). The *Junbo* mice used in our study were genetically identical and raised under SPF conditions, suggesting that inherent and environmental factors might not be significantly contributing to this variability. Examination for other bacteria in the non‐NTHi inoculated *Junbo* mouse middle‐ear fluid using different culture media and conditions showed low or no bacterial load. Inoculation of NTHi increased the load of other culturable bacteria in the middle‐ear fluid but was not significantly variable across different grades of fluid and did not correlate with the infection of NTHi (data not shown). This suggests that NTHi was the major contributor to the cellular variation that we observed, but an influence of nonculturable bacteria cannot be ruled out; high‐throughput microbiome analysis is currently being undertaken. One other noteworthy observation in our study was that even in the same mouse, differences in the middle‐ear fluid and NTHi infection were frequently observed, indicating that the variability is contained within the middle ear environment and might not be due to the systemic differences (if any) in individual mice; currently these and other factors are under investigation. By exploring the underlying mechanism, we will gain insight into the reasons for the variable chronicity of the OM and NTHi infection, and consequently, this could inform us on novel targets for prophylaxis.

## 4 EXPERIMENTAL PROCEDURES

### 4.1 Ethics statement

The humane care and use of mice in this study was carried out under the appropriate U.K. Home Office licence, and the experiments were passed by the local ethics review committee.

### 4.2 Mice and bacterial strains

The heterozygote *Junbo* mouse (*Jbo/+,* hereafter termed *Junbo*) was congenic on a C3H/HeH background. The mice were specific pathogen free and had normal commensal flora. Nontypeable *Haemophilus influenzae* (NTHi) strain 162Sr (streptomycin resistant; Hood et al., 2016) was grown in brain heart infusion (BHI) media supplemented with Leventhal's reagent. Prior to intranasal inoculation, NTHi 162Sr was washed with phosphate*‐*buffered saline (PBS) then resuspended in PBS‐2% gelatine (Hood et al., 2016).

### 4.3 *Junbo* mouse infection with NTHi

The inoculation of the *Junbo* mice was carried out as described previously (Hood et al., 2016). Briefly, *Junbo* mice were inoculated intranasally under gas anaesthesia with 5 μl per nares of NTHi 162 sr cell suspension at a concentration of 1 × 10^8^ cfu/ml in PBS‐2% gelatine. Bulla fluid was collected at seven‐day postinoculation from mice under terminal anaesthesia induced by an intraperitoneal overdose of sodium pentobarbital. After removal of any material on the external surface of the tympanic membrane, a hole was made in the membrane whilst removing the malleus from the middle ear using a sterile pair of forceps. Bulla fluid was collected with a pipette and microtip; the bulla fluid was graded under the microscope on the basis of its viscosity and opacity during collection. Bulla fluid volume was measured by collecting up to 0.5 μl aliquots multiple times, and the total volume generally ranged between 0.25 and 1.5 μl. Bulla fluid was collected into 10 μl PBS buffer, and a portion of it was serially diluted in PBS and plated on BHI agar supplemented with streptomycin (300 μg/ml); the bacterial titre was calculated from the colony count following overnight growth of the plates. The remainder of the fluid was used for flow cytometry and cytokine/chemokine analyses.

### 4.4 Flow cytometry analysis of bulla fluid

Bulla fluids with different viscosity indices were diluted with flow cytometry (FACS) buffer (5 mM EDTA, 0.5% foetal calf serum in PBS) to a final volume of 200 μl, and a cell count was obtained. The bulla fluids were transferred to a 96 well plate at the concentration of 2 × 10^5^ cells/well. Cells were centrifuged and washed twice with FACS buffer. To analyse the apoptotic, live, and necrotic cell content, the BioLegend's FITC Annexin V Apoptosis Detection Kit with propidium iodide was used. For the analysis of different types of immune cells, the suspension was incubated for 15 min with CD16/CD32 antibody (BD Pharmagen™) at a dilution of 1:100. After centrifugation at 800 g for 1 min, cell pellets were resuspended in 100 μl of antibody cocktail {F4/80–PE (1:200; e biosciences™), CD11b – PE – CF594 (1:200), Ly6G – BV421 (1:200), Ly6C – FITC (1:200) and CD5 – BD421 (1:800; BD Horizon™)} and incubated for 20 min in the dark. Cells were centrifuged and washed twice with FACS buffer and finally resuspended in 100 μl of Sytox DNA stain (1:10,000). After making up the final volume to 250 μl with FACS buffer, FACS was performed using a BD FACSCanto™ II system. FlowJo software (Tree Star™) was used to analyse the data obtained.

### 4.5 Histology and immunohistochemistry

Middle‐ear histology sections and Haematoxylin and Eosin staining of *Junbo* mice heads were carried out as previously described (Cheeseman et al., 2011). To examine the neutrophil and NTHi content by immunohistochemistry, rabbit antimyeloperoxidase (anti‐MPO, Abcam™) and anti NTHi‐162 antibodies were used. Anti‐NTHi 162 Sr sera was raised in the rabbit (Covalab); this and control non‐immune (prebleed) serum were purified further by affinity chromatography using the Protein G Sepharose®, Fast Flow beads (Sigma) as per the manufacturer's instructions. The purified antibody was assessed by western blot and immunostaining. Unstained histology slides with 4‐μm thick samples were dewaxed using xylene. Endogenous peroxidase was blocked using peroxidase blocker (Dako REAL S2023) for 10 min following antigen retrieval by two intervals of heating in a microwave for 7 min in distilled water. Secondary antibody binding was carried out using the rabbit VERTEX ABC kit. Visualisation was achieved using Dako (K3468) liquid DAB+ substrate chromogen system. Counterstaining was carried out using Harris Haematoxylin and mounting in Clearview mounting (Thermo Fisher Scientific). The slides were visualised using a Hamatasu™ NDP Nanozoomer.

### 4.6 Cytokine and chemokine responses

Cytokine and chemokine levels were measured in bulla fluids collected from NTHi‐infected *Junbo* mice (*n* = 4) using a mouse cytokine antibody array (Mouse Cytokine Array C1, RayBiotech Inc.™) according to the manufacturer's protocol. The following cytokines and chemokines were analysed: GCSF, GMCSF, interleukins (IL); IL‐2, IL‐3, IL‐4, IL‐5, IL‐6, IL‐9, IL‐10, IL‐12p40/p70, IL‐12p70, IL‐13, IL‐17 as well as interferon gamma (IFN‐γ), monocyte chemoattractant proteins (MCP: MCP‐1, MCP‐5), T cell expressed and secreted (RANTES), stem cell factor (SCF), soluble tumour necrosis factor (TNF) receptor type I (sTNFRI), TNF‐α, thrombopoietin (THPO), and the vascular endothelial growth factor (VEGF). The signal intensities were quantified using the Image Lab™ Software (BioRad). Changes in CCL2, CCL3, CXCL2, and CXCR2 were analysed by direct ELISA. Diluted *Junbo* mouse bulla fluid or serum was added to the high affinity 96 well plates (Thermo Scientific™) and allowed to bind by incubating overnight at 4°C. The wells were washed three times with PBS and primary antibodies against respective chemokines obtained from the R&D systems™ (CCL2‐AF479‐NA, CCL3‐MAB450, CXCL2‐AF452‐NA, CXCR2–MAB2164) were added and incubated for 2 hr at room temperature with shaking. The wells were washed three times with PBS and respective HRP conjugated secondary antibodies were added. After incubation for 1 hr at room temperature with shaking, the wells were washed three times with PBS, and 100 μl of TMB substrate (Thermo Scientific™) solution was added. The plate was incubated for 15 min at room temperature, and the reaction was stopped by adding 2 N H_2_SO_4_. The colour change was measured at 450 nm on an Epoch Biotek™ plate reader and fold difference with respect to either NTHi−ve MEF or noninoculated *Junbo* mouse serum was calculated.

## CONFLICT OF INTEREST

The authors declare no conflict of interest.

## AUTHOR CONTRIBUTIONS

P. Vikhe, T. Purnell, and D. Hood designed and conducted experiments along with data analyses. PV, DH and S. D. Brown guided the work and contributed to manuscript writing and results evaluation.

## FUNDING INFORMATION

This work was funded by the Medical Research Council, (MRC funding award no. MC_U142684175).

## Supporting information

Data S1 Supporting Information
